# Identification of a Novel Long Non-Coding RNA G8110 That Modulates Porcine Adipogenic Differentiation and Inflammatory Responses

**DOI:** 10.3390/ijms242316799

**Published:** 2023-11-27

**Authors:** Jin Chai, Ning Wang, Li Chen, Jingyi Bai, Jiaman Zhang, Geng Zhang, Jiahua An, Tingting Zhang, Xingyan Tong, Yifan Wu, Mingzhou Li, Long Jin

**Affiliations:** 1 State Key Laboratory of Swine and Poultry Breeding Industry, College of Animal Science and Technology, Sichuan Agricultural University, Chengdu 611130, China; cj1013713817@163.com (J.C.); wn1025035235@163.com (N.W.); jingyi_bai@163.com (J.B.); zjm960521@163.com (J.Z.); zg1309465843@163.com (G.Z.); 19382119267@163.com (J.A.); ztt18364732158@163.com (T.Z.); 17361123793@163.com (X.T.); wuyifan2000lw@163.com (Y.W.); 2 Key Laboratory of Livestock and Poultry Multi-Omics, Ministry of Agriculture and Rural Affairs, College of Animal Science and Technology, Sichuan Agricultural University, Chengdu 611130, China; 3 Chongqing Academy of Animal Science, Chongqing 402460, China; lichen5696@163.com; 4 National Center of Technology Innovation for Pigs, Chongqing 402460, China; 5 Key Laboratory of Animal Resource Evaluation and Utilization (Pigs), Ministry of Agriculture and Rural Affairs, Chongqing 402160, China; 6 Key Laboratory of Agricultural Bioinformatics, Ministry of Education, Sichuan Agricultural University, Chengdu 611130, China

**Keywords:** lncRNA, adipogenesis, lipid metabolism, inflammation

## Abstract

Long non-coding RNAs (lncRNAs) have been extensively studied, and their crucial roles in adipogenesis, lipid metabolism, and gene expression have been revealed. However, the exact regulatory or other mechanisms by which lncRNAs influence the functioning of mesenteric adipose tissue (MAT) remain largely unknown. In this paper, we report the identification of a new lncRNA, named G8110, from the MAT of Bama pigs. The coordinated expression levels of lncRNA G8110 and *NFE2L1* were significantly decreased in the MAT of obese Bama pigs compared with those in the MAT of lean pigs. Using a bone mesenchymal stem cell adipogenic differentiation model, we found that lncRNA G8110 played a role in adipocyte differentiation by positively regulating *NFE2L1*. We also found that lncRNA G8110 inhibited the formation of intracellular lipid synthesis, promoted lipid metabolism, and inhibited the expression of inflammatory cytokines. Our findings regarding lipid synthesis may further promote the role of lncRNAs in driving adipose tissue remodeling and maintaining metabolic health.

## 1. Introduction

Obesity has become a global public health hazard and is the leading cause of cardiovascular disease, type 2 diabetes, and obesity-related metabolic syndrome [1,2]. As much as 58% of the world’s adult population is predicted to be overweight or obese by 2030 [2]. The metabolic risk factors for obesity are more closely related to adipose distribution than to total adipose mass, possibly because of significant differences in the roles of anatomically distinct adipose tissues (ATs) in maintaining energy balance [3]. In individuals with obesity, the risk of diabetes and cardiovascular disease is greater when excess energy is stored in visceral ATs (VATs) than in subcutaneous ATs (SATs) [4]. Mesenteric AT (MAT) is a component of VAT, and hypertrophic MAT is associated with the development of inflammatory bowel diseases (IBDs) [5].

Obesity is closely associated with many regulators of adipogenesis, including coding and non-coding genes. Long non-coding RNAs (lncRNAs) are composed of more than 200 nucleotides and are extensively involved in epigenetic, transcriptional, and post-transcriptional regulation in cells [6,7,8,9,10,11]. In recent years, the application of transcriptome sequencing has eased the identification of a large number of novel lncRNAs in AT [12,13]. A growing number of studies have shown that lncRNAs are key regulators of adipocyte differentiation, insulin signaling, and white adipose tissue browning (WAT). For example, adiponectin AS lncRNA inhibits adipogenesis by transferring from the nucleus to the cytoplasm and attenuating adiponectin mRNA translation. The lncRNA Blnc1 promotes brown and beige adipocyte differentiation and function [8,14,15,16]. Although numerous studies have demonstrated the role of lncRNAs in adipogenesis [17,18], their functional roles and regulatory mechanisms remain unclear. 

The physiological structure, organ sizes, and metabolic characteristics of pigs are highly similar to those of humans [19,20]. In particular, pigs exhibit regional variations in anatomically discrete adipose depots (e.g., SATs and VATs) that are highly similar to humans, highlighting the value of pigs for use in obesity research models.

In this study, we identified a novel lncRNA, which we named G8110, from the MAT of Bama pigs. Our predictive analysis revealed the regulation of *NFE2L1* as a possible target of G8110. Additionally, the expression levels of G8110 and *NFE2L1* were highly positively correlated in 48 Bama pigs (12 pigs from the normal conditions [NC] group and 36 from the weight gain [WG] group) [21]. By establishing an adipogenic differentiation model using mesenchymal stem cells (BMSCs) from Bama pig bones, we found that G8110 plays a role in adipogenic differentiation by positively regulating *NFE2L1*. This regulatory relationship plays an important role in weight gain in Bama pigs. Notably, we also found that G8110 promoted biological processes such as intracellular oxidative phosphorylation and glycolipid metabolism and inhibited the generation and secretion of inflammatory adipokines, which might represent an adaptive mechanism to promote the healthy remodeling of AT and maintain metabolic homeostasis during obesity. This study illustrates the role of lncRNAs in orchestrating adipose adaptation to obesity and maintaining systemic metabolic health, not only expanding the network of adipogenesis-related regulatory lncRNAs but also providing potential molecular therapeutic targets for the treatment of obesity-related diseases.

## 2. Results

### 2.1. Identification and Functional Prediction of lncRNA G8110

**Figure 1:**
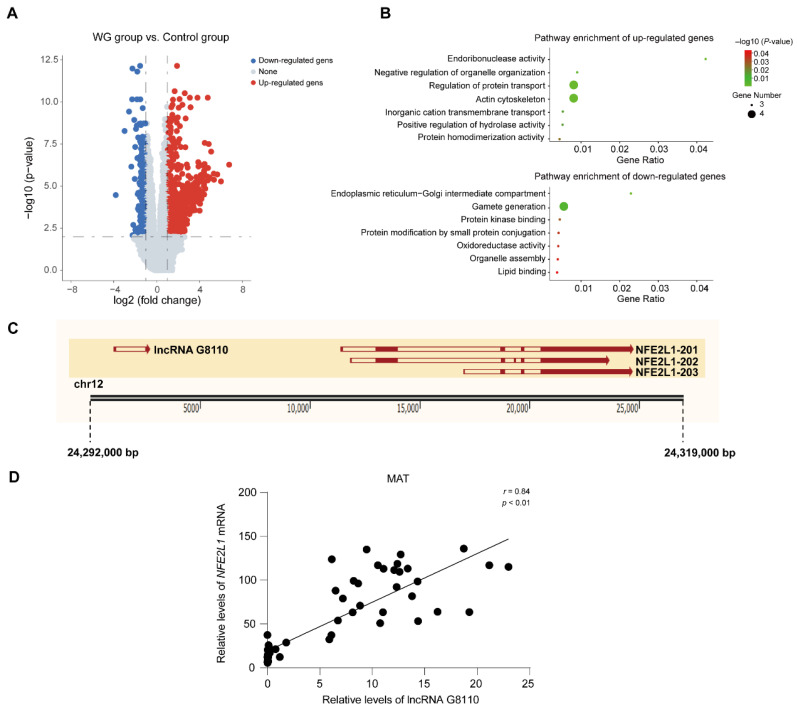
The identification of lncRNA G8110 and the prediction of its regulatory target genes. (**A**) Volcano plot of differentially expressed genes. Positive values indicate up-regulation; negative values indicate down-regulation. The *x*-axis represents the log2 scale of fold change. A significant difference in expression is indicated by the *y*-axis, which displays the −log10 scale of adjusted *p* values. (**B**) GO and KEGG pathway analysis of the target genes of lncRNAs with significant differential expression. Only the pathways with a *p* < 0.05 after correction are listed. (**C**) A schematic diagram of the positions of *NFE2L1* and lncRNA G8110 on chromosome 12. (**D**) Pearson’s correlation analysis between *NFE2L1* and lncRNA G8110 mRNA expression levels in the MAT of 48 Bama pigs. Data are expressed as the mean ± SEM.

We used published RNA sequencing (RNA-seq) data from a previous study [21] to quantify the lncRNA expression profiles of MAT between Bama pigs within a normal condition (NC, *n* = 12) group (healthy pigs fed a normal diet) and a weight gain (WG, *n* = 36) group (pigs fed according to a high-fat diet). A total of 19,112 lncRNAs were identified (19,112 lncRNAs from previous annotations) [21], and the results are displayed in Table S1. To explore the potential biological functions of lncRNAs in the mesenteric adipose tissue of Bama pigs, we identified the lncRNAs that were differentially expressed between the two groups. A total of 1395 differentially expressed lncRNAs were screened, and in the WG group, 1132 lncRNAs were up-regulated and 262 lncRNAs were down-regulated. In this regard, a comparison between the WG group and the NC group is shown in Figure 1A. We pinpointed highly expressed (TPM > 2) lncRNAs and found that 9 lncRNAs were significantly up-regulated and that 15 lncRNAs were significantly down-regulated (Table S2).

Next, we detected neighboring (100 Kb upstream and downstream of lncRNAs) and expression-correlated protein-coding genes (PCGs) for these significantly differentially expressed lncRNAs (DE lncRNAs). We identified 42 (Table S3) and 78 (Table S4) lncRNA-protein-coding gene pairs for up-regulated and down-regulated DE lncRNAs, respectively. Then, in order to dissect the biological functions of these DE lncRNAs, we performed a functional enrichment analysis on associated PCGs (Figure 1B). We found that the target PCGs of the up-regulated lncRNAs were mainly enriched in pathways such as endoribonuclease activity, regulation of protein transport, and inorganic cation transmembrane transport; the target PCGs for the down-regulated lncRNAs were mainly enriched in protein kinase binding, oxidoreductase activity, lipid binding, and other pathways. 

Of these genes, the *NFE2L1* gene, which is located in the neighborhood of lncRNA G8110 (located 8656 bp upstream of *NFE2L1*) (Figure 1C), plays an important role in metabolism and lipid binding [22,23]. Furthermore, we calculated the Pearson’s correlation coefficients of lncRNA G8110 and *NFE2L1* expression across 48 Bama pigs (12 NC group pigs and 36 WG group pigs) [21] and found a highly positive correlation (*r* = 0.84, *p* < 0.01), suggesting the potential regulatory role of lncRNA G8110 in regulating the expression of *NFE2L1* (Figure 1D). Taken together, these results suggest a potential collaboration between lncRNA G8110 and the *NFE2L1* gene in adipogenesis in pigs with weight gain induced by high-fat diet feeding.

### 2.2. LncRNA G8110 Positively Regulates the Expression of NFE2L1

**Figure 2:**
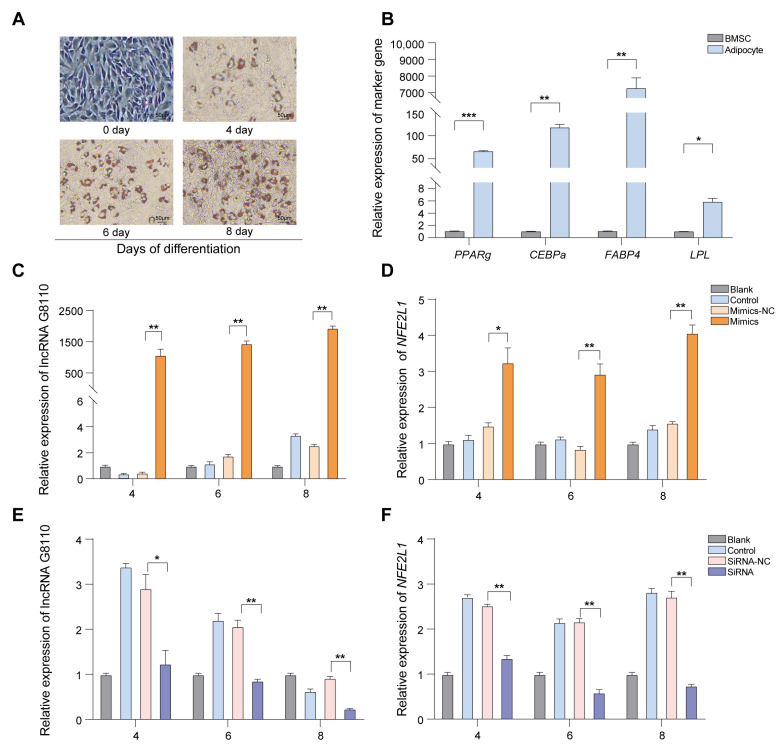
Effect of overexpression and knockdown of lncRNA G8110 on *NFE2L1*. (**A**) Establishment of a BMSC adipogenic differentiation model. Oil-red O staining of Bama pig BMSCs at days 4, 6, and 8 of adipogenic induction into adipose cells. Scale bar: 50 μm. (**B**) To confirm the successful differentiation of the BMSCs into adipose cells, RT-qPCR was used to detect the expression of adipogenic marker genes in the BMSCs at 8 days of adipogenic differentiation. (**C**) Measurement of the lncRNA G8110 overexpression level at various time points after the adipogenic differentiation of the BMSCs. (**D**) Expression levels of *NFE2L1* mRNA in BMSCs under the overexpression of lncRNA G8110 at various time points after adipogenic differentiation. (**E**) Measurement of the expression levels of lncRNA G8110 under siRNA-mediated knockdown at various time points after the adipogenic differentiation of the BMSCs. (**F**) Measurement of *NFE2L1* mRNA expression under lncRNA G8110 knockdown at various time points after the adipogenic differentiation of the BMSCs. Note: Expression levels were standardized to the expression level of the gene before the induction of differentiation. Data are expressed as the mean ± SEM; * *p* < 0.05, ** *p* < 0.01, *** *p* < 0.001.

Using the established BMSC adipogenic differentiation model (Figure 2A,B), we further investigated the regulatory role of lncRNA G8110. We constructed an overexpression vector for lncRNA G8110 (Figure S1), which was transfected into BMSCs under lipogenesis induction. Our RT-qPCR results showed that lncRNA G8110 was successfully transfected at days 4, 6, and 8 of the adipogenic differentiation of the BMSCs (Figure 2C). The relative expression of *NFE2L1* under lncRNA G8110 overexpression increased significantly at these three time points (Figure 2D).

Next, we performed a knockdown assay to down-regulate lncRNA G8110 during the adipogenic differentiation of the BMSCs. We found that the expression of lncRNA G8110 was down-regulated via transfection with small-interfering RNAs (siRNAs) targeting it (Figure 2E). The mRNA expression level of *NFE2L1* was significantly decreased under the knockdown of lncRNA G8110 during the adipogenic differentiation of the BMSCs (Figure 2F). Based on these results, we speculated that lncRNA G8110 positively regulated the expression of *NFE2L1*, thus playing a role in lipid synthesis and adipocyte differentiation.

### 2.3. LncRNA G8110 Inhibited the Adipogenic Differentiation of BMSCs

**Figure 3:**
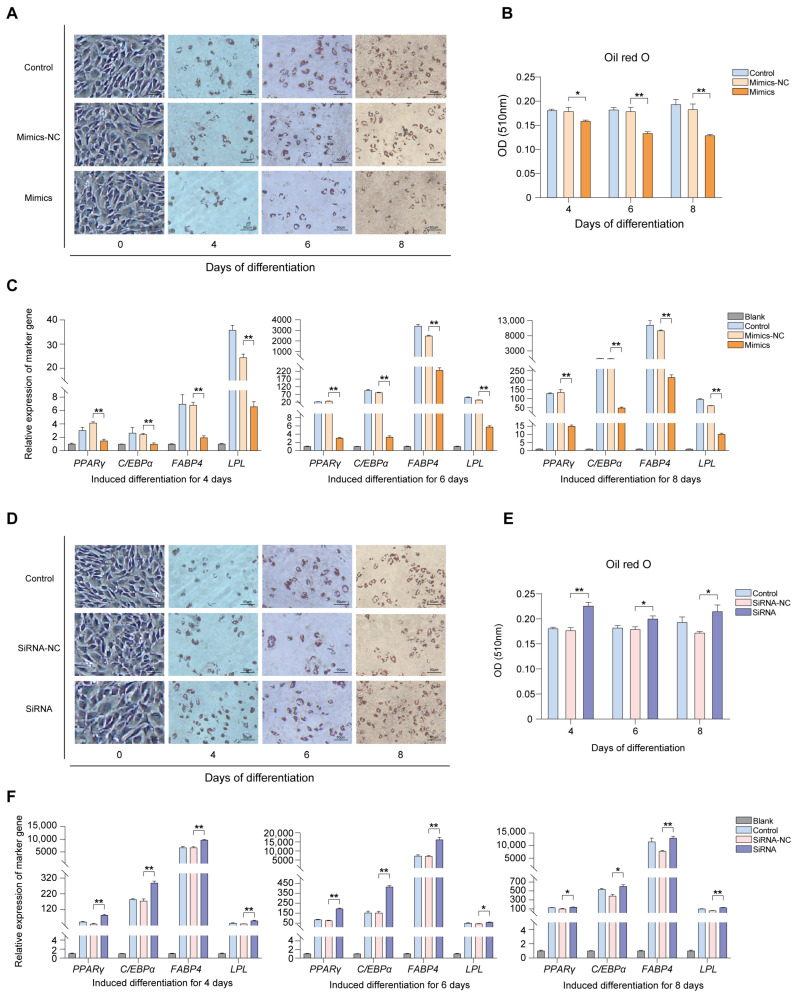
Lipid droplet formation and expression of lipid marker genes after the adipogenic differentiation of BMSCs under lncRNA G8110 overexpression or knockdown. (**A**) Oil red O staining of adipocytes at days 4, 6, and 8 of BMSC adipogenesis after the overexpression of lncRNA G8110 (200×, bright-field shooting). (**B**) Quantitative detection of oil red O staining in terms of OD; the figure shows significantly lower values in the Mimics group than in the empty vector group. (**C**) Measurement of adipogenic marker gene expression in BMSCs with an overexpression of lncRNA G8110 at various time points after adipogenic differentiation. (**D**) Oil red O staining of adipocytes at days 4, 6, and 8 of adipogenic differentiation after lncRNA G8110 knockdown (200×, bright-field shooting). (**E**) Quantitative detection of oil red O staining. (**F**) Measurement of the expression of adipogenic marker genes in BMSCs under lncRNA G8110 knockdown at various time points after the adipogenic differentiation of the BMSCs. Note: The expression level was standardized to the expression level of the gene before cell differentiation. Data are expressed as the mean ± SEM; * *p* < 0.05, ** *p* < 0.01.

To further examine the effect of lncRNA G8110 on adipogenic differentiation, we transfected Bama pig-derived BMSCs with the lncRNA G8110 overexpression vector or the empty vector and then induced adipogenic differentiation. To observe the phenotypic effect of lncRNA G8110 overexpression on adipogenic differentiation, we stained the cells in each treatment group with oil red O and examined their triglyceride contents (reflected by OD values) (Figure 3A). The size and number of intracellular lipid droplets were significantly lower in cells under lncRNA G8110 overexpression than in the control cells at each of the differentiation time points (Figure 3B). Then, the expression levels of four adipogenic marker genes (*PPARγ, C/EBPα, FABP4*, and *LPL*) at various adipogenic induction times were measured using RT-qPCR, and the results showed that the overexpression of lncRNA G8110 significantly decreased the expression levels of these adipogenic markers on days 4, 6, and 8 of cell differentiation (Figure 3C).

We also performed siRNA interference experiments. Similarly, oil red O staining showed that the triglyceride content of the cells transfected with siRNA was significantly higher than that in the cells transfected with siRNA-NC at the three differentiation time points (Figure 3D,E). These results indicated that the knockdown of lncRNA G8110 increased lipid droplet accumulation. Consistently, the expression levels of all four adipogenic marker genes (*PPARγ*, *C/EBPα*, *FABP4*, and *LPL*) significantly increased after lncRNA G8110 knockdown during cell differentiation (Figure 3F). These results suggest that lncRNA G8110 can inhibit the adipogenic differentiation of BMSCs.

### 2.4. LncRNA G8110 Promotes Lipid Metabolism in Adipocytes and Affects Inflammation and Mitochondrial Function

Previous studies have revealed that *NFE2L1* deficiency is associated with decreased lipolysis ability, cellular inflammation, and the mitochondrial respiratory function of adipose tissue [24,25,26]. To examine the effects of lncRNA G8110 on these functions of adipose cells, we used RT-qPCR to measure the expression of representative lipolysis genes, cellular inflammation-related genes, and mitochondrial marker genes in cells under lncRNA G8110 overexpression or interference.

**Figure 4:**
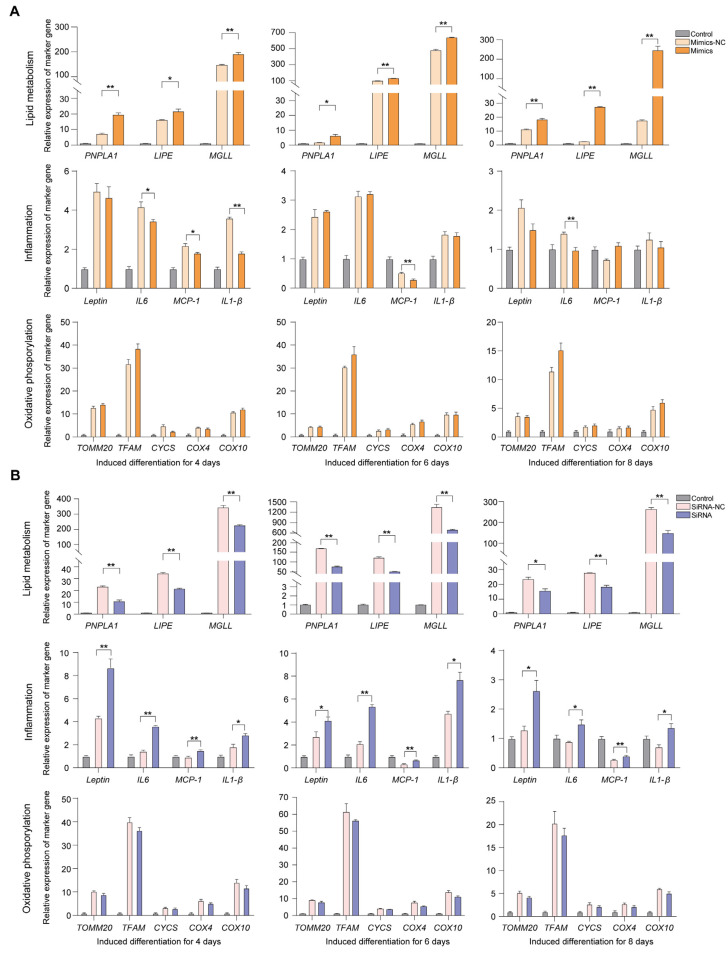
LncRNA G8110 promotes lipid metabolism in adipose tissue and affects biological processes. (**A**,**B**) Measurement of the expression levels of genes related to lipid metabolism, cellular inflammation, and mitochondria in BMSCs with an overexpression of G8110 (**A**) or knockdown of G8110 (**B**) at various time points after adipogenic differentiation. Note: Expression levels were standardized to the expression level of the gene before cell differentiation. Data are expressed as the mean ± SEM; * *p* < 0.05; ** *p* < 0.01.

The results showed that the expression levels of three lipolysis genes (*PNPLA1*, *LIPE*, and *MGLL*) in the overexpressed group were significantly up-regulated compared with the empty vector group on days 4, 6, and 8 of the adipogenic differentiation of the BMSCs. At these three time points, the expression levels of cellular inflammation marker genes (i.e., *Leptin*, *IL6*, *MCP-1*, *IL1-β*) and mitochondrial marker genes (i.e., *TOMM20*, *TFAM*, *CYCS*, *COX4*, *COX10*) demonstrated increases but no significant changes (Figure 4A), which is consistent with the above experimental results.

Under the knockdown of lncRNA G8110, the expression levels of the three lipolysis genes were significantly down-regulated, and those of the four cellular inflammation-related genes were significantly up-regulated at all three time points. The five mitochondrial marker genes exhibited decreases in their expression levels but were not significantly changed (Figure 4B). These results indicated that lncRNA G8110 played a positive role in promoting lipid metabolism and the oxidative decomposition of adipocytes during the adipogenic differentiation of the BMSCs, inhibiting the expression of inflammatory adipokines.

## 3. Discussion

Using high-throughput sequencing [27,28], an increasing number of lncRNAs have been identified as important players in adipogenic differentiation and the development of AT, regulating the expression of adipogenic marker genes such as *PPARγ* and *SREBF1* [29,30,31]. *NFE2L1* (also known as *NRF1*), which belongs to the *CNC-bZIP* family, is a major negative regulator of lipid synthesis, and it may play a crucial role in energy metabolism [22,23]. In this paper, we have reported the identification of a new lncRNA, G8110, from the MAT of Bama pigs. LncRNA G8110 is located near the upstream regulatory region of *NFE2L1,* and the expression of the two showed a positively correlated expression in the BMSCs, with lncRNA G8110 up-regulating the transcription of *NFE2L1*. Furthermore, the expression levels of lncRNA G8110 and *NFE2L1* in the MAT of obese Bama pigs were both significantly decreased compared to those of lean pigs. Moreover, by establishing an adipogenic differentiation model based on Bama pig-derived BMSCs, we found that lncRNA G8110 plays a role in lipid differentiation by positively regulating *NFE2L1*. We also constructed a network centered on lncRNA G8110 (Figure S2). The protein-protein interaction network of these tested genes (the tested genes in Figure 3 and Figure 4) is shown in Figure S3. We found fewer interactions in these PCGs that have been experimentally determined, and their relationships remain to be further experimented with and explored.

Recently, a large number of studies on the effect of lncRNAs on adipogenesis in human and animal models have provided us with a mechanistic understanding of adipose deposition and lipid metabolism in animals, as well as insights into lipid-related diseases. For example, lncRNA RP11-142A22.4 regulates Wnt5β expression by sponging miR-587 in human preadipocytes to control adipocyte differentiation [32]. The lncRNA Plnc1 may reduce the methylation level of the CpG region in the PPAR-g2 promoter to enhance its transcriptional activity, thereby increasing PPAR-g2 transcription and promoting adipogenic differentiation [31]. Meanwhile, in pigs, for instance, *PU.1* antisense lncRNA transcribed from porcine *PU.1* inhibits the translation of *PU.1* mRNA and promotes adipose production [33]. The lncRNA *PLA2G16*-AS regulates the expression of *PLA2G16* by complementing the host gene, which plays an important role in porcine fat deposition [34,35]. In this paper, we explore the potential molecular mechanism of lncRNA G8110 in regulating adipocyte differentiation. LncRNA G8110 is located 8656 bp upstream of *NFE2L1*, in the predicted enhancer region. Enhancers feature special markers of chromatin modification, and one of the main features of enhancer transcription is the presence of the chromatin histone modification H3K27ac [36,37,38,39,40,41]. By analyzing the corresponding H3K27ac ChIP-Seq data (WG, *n* = 3), we found enriched H3K27ac signals in the region of lncRNA G8110 (Figure S4). Activated enhancers recruit coactivators and transcription factors (TFs) to produce enhancer RNAs (eRNAs) [42], which, together with TFs and RNA-binding proteins (RBPs), can mediate the expression of neighboring genes [43,44,45]. We found an enrichment of binding motifs for adipogenic TFs such as *TBX1* and *TFAP2B* in the lncRNA G8110 region [46,47,48,49] (Figure S5). Considering the information above, we speculate that lncRNA G8110 may act as an eRNA to regulate the transcriptional activity of target genes. However, the mechanism of lncRNA G8110 in such a role needs to be studied further.

**Figure 5:**
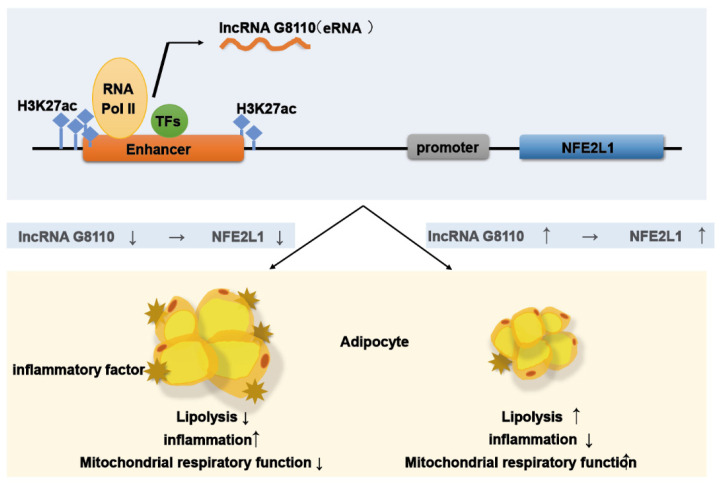
Schematic diagram of the possible regulatory mechanism of lncRNA G8110 in its regulation of adipose tissue.

In recent decades, numerous animal model studies have significantly improved our understanding of the pathogenesis of obesity and its associated diseases in humans [20]. Some studies have found that the pathogenesis of SAT and VAT in obesity in pigs is similar to that in humans [50]. Therefore, pigs have gradually become a key point of focus in metabolic research. AT stores energy and regulates endocrine functions [51]. Previous studies have found that VAT exhibits macrophage infiltration and inflammatory responses that are strongly associated with metabolic syndrome [52]. MAT is a unique visceral fat with certain anatomical properties, acting as a barrier with immune, antibacterial, and endocrine functions [53]. For example, Crohn’s disease is an IBD [54], primarily of the small bowel, that presents with hyperplastic MAT, which expands and wraps specifically around sites of intestinal inflammation [55]. Previous studies have indicated that AT accumulation in the mesentery is accompanied by inflammation. Although there are a limited number of studies that explore *NFE2L1* expression as a direct cause of Crohn’s disease, its typical genetic association and functional significance in other IBDs suggest that it may play an important role [56,57]. The expression levels of lncRNA G8110 and *NFE2L1* were decreased in the WG group, and lncRNA G8110 showed beneficial effects by promoting lipid metabolism and inhibiting adipose inflammation (Figure 4). Excessive ectopic lipid accumulation in recruited macrophages and other immune cells promotes systemic inflammation and upholds ectopic lipid accumulation [58,59,60]. At the same time, the development of associated metabolic syndromes (e.g., insulin resistance) [61] in individuals with obesity is accompanied by excessive cellular oxidative stress, oxidative damage to mitochondrial components, and a reduced capacity for oxidative phosphorylation [62,63]. Thus, our results provide new clues to guide investigations of the abnormal accumulation of visceral fat (Figure 5). Experimental studies on other species (e.g., mice and non-human primates) are needed to functionally validate the biological mechanisms of the key genes and their regulatory elements that underpin complex traits and diseases.

## 4. Materials and Methods

### 4.1. Ethics Statement

All research procedures involving animals were carried out according to the Regulations for the Administration of Affairs Concerning Experimental Animals (Ministry of Science and Technology, China, revised in March 2017) and approved by the animal ethical and welfare committee (AEWC) of Sichuan Agricultural University under permit DKY-2021302166.

### 4.2. Porcine Bone Mesenchymal Stem Cell Isolation

A one-month-old female Bama pig was sacrificed to harvest hind leg bones without muscle or fascia. A 25 g needle was used to make holes at both ends of the bone. Next, a 20 mL syringe containing serum-free medium (Lonza, Hayward, CA, USA) with serum substitutes (Pall, San Diego, CA, USA) was used to rinse the bone and obtain the marrow. Bone marrow was passed through a 40 μm filter (FALCON, Warren, NJ, USA). The washed whole bone marrow stem cells were transferred from the culture medium to a centrifuge tube and spun via centrifugation. The supernatant was discarded, and red blood cell lysate was added and allowed to stand for 5 min before the cells were pelleted through centrifugation at 1000× *g* for 4 min at room temperature. The cells were resuspended, plated in T75 cell culture flasks (Corning, CA, USA), and cultured in a 5% CO_2_ incubator at 37 °C. Adherent cells remaining 4 h later were considered bone marrow mesenchymal stem cells. These cells were frozen, resuscitated, and passaged up to 18 times.

### 4.3. Cell Transfection and Adipogenic Differentiation 

For the lncRNA G8110 overexpression and knockdown assays, cells were seeded in 24-well plates. At 80–90% confluence, the lncRNA G8110 plasmid (the full-length sequence of lncRNA G8110 was inserted into the plasmid), the pEGFP-N1 empty plasmid, a small interfering RNA (siRNA), and a siRNA-negative control were added (Table S5). For the overexpression of lncRNA, cells were divided into BMSCs (Blank), a normal adipogenic differentiation group (Control), an empty vector group (Mimics-NC), and an overexpression group (Mimics). For the interference experiments, cells were divided into BMSCs (Blank), a normal adipogenic differentiation group (Control), a negative control group (siRNA-NC), and a knockdown group (siRNAs). Cells were transfected using Lipofectamine 3000 (Invitrogen, Carlsbad, CA, USA) following the manufacturer’s instructions (with a vector concentration of 500 ng per well). After transfection for 24 h, the culture medium was removed; adipogenic differentiation medium A (Cyagen, Guangzhou, China) was added for 2 days and then replaced with adipogenic induction maintenance medium B (Cyagen) for 2 days. After two cycles (8 days in total), adipose cells containing large, round lipid droplets had appeared. The cells were differentiated for 4, 6, and 8 days, collected, and stored at −80 °C for further analysis.

### 4.4. Oil, Red O Staining, and Triglyceride Content Detection

After adipogenic induction, the cells were washed two or three times with phosphate-buffered saline (PBS) before being fixed in 1 mL of 4% paraformaldehyde for 30 min. After rinsing twice with 60% isopropanol and air drying, a 1 mL working solution of oil red O dye was added for 30 min. Then, 1 mL of PBS was added to the culture plate, and the oil red O staining was observed under a microscope. To measure their triglyceride contents, the stained adipose cells were washed with deionized water, isopropanol was added to elute the oil red O dye, and absorbance was measured on a microplate reader (Thermo Varioskan LUX, Waltham, MA, USA) at 510 nm.

### 4.5. Real-Time Quantitative PCR

In accordance with the manufacturer’s protocol for HiScript III RT SuperMix in qPCR (Vazyme, Nanjing, China), total RNA was reverse-transcribed to cDNA using Oligo-dT and random hexamers. RT-qPCR was carried out using the Taq Pro Universal SYBR qPCR Master Mix (Vazyme). The primers used for RT-qPCR were designed using Primer (version 5.0) software, and the sequences are shown in Table S6. RT-qPCR was performed on a CFX96 Real-Time PCR detection system (Bio-Rad, Hercules, CA, USA); the thermal cycle conditions used in the RT-qPCR reaction are shown in Table S7. Each sample was analyzed in triplicate. The relative expression levels of the tested genes were calculated using the 2^−ΔΔCT^ method, with porcine glyceraldehyde-3-phosphate dehydrogenase (*GAPDH*) and β-actin (*ACTB*) serving as internal controls.

### 4.6. Data Analysis

All annotated protein-coding genes (PCGs) in the reference genome (Sscrofa11.1, release 102), together with long non-coding RNA (lncRNA) transcript annotations from our previous study [50], were utilized for gene annotation and subsequent transcript quantification. Gene-level expression was estimated as transcripts per million (TPM) using the high-speed transcript quantification tool Kallisto (version 0.43.0) with parameters (--bias --rf-stranded). Mapped read counts for each gene were obtained by employing the tximport (version 1.6.0) R package with default settings. We considered a PCG to be detected/expressed if its expression level was greater than 0.5 TPM in at least one sample. For lncRNAs, we used a cutoff of 0.1 TPM in at least one sample.

Based on the counts of each lncRNA, differential lncRNA expression analysis was performed using edgeR (version 3.22.5) [64], with a false discovery rate (FDR) ≤ 0.01 and log2(fold change) ≥ 1 as the cutoffs for statistical significance.

The prediction of lncRNA function was based on PCGs located near the lncRNA and correlated with expression levels. The distance parameter in this study was set at 100 Kb (Kilobase), that is, all coding genes within 100 Kb upstream and downstream of the lncRNA genome position were extracted.

Candidate target genes were first converted to their human orthologs and used as inputs, taking all the annotated PCGs in the reference human genome as the background. Next, the obtained homologous genes were uploaded to the Metascape web server (https://metascape.org) for gene ontology (GO) analysis, including the molecular function and biological process categories, and Kyoto Encyclopedia of Genes and Genomes (KEGG) pathway analysis. Genes that exhibited a Benjamini-adjusted *p* value < 0.05 for a given pathway were considered to be significantly enriched in that pathway.

We used published Chromatin immunoprecipitation sequencing (ChIP-seq) data from a previous study and analyzed these data according to the method therein [21]. Briefly, H3K27ac ChIP-seq reads were mapped to the pig reference genome (Sscrofa 11.1) using BWA110 (version 0.7.15) [65]. Next, PCR duplicates were removed using Samtools 111 (version 1.3.1) [66]. Peaks were called using the SICER112 tool (version 0.1.1) [67] with the following parameters: (--windowSize 200 --gapSize 3 --mapq 0 --fragSize 250 --FDR 0.05). Fold enrichment over control signal tracks was determined using the bdgcmp command in MACS2 (version 2.1.1.20160309) with default parameters.

Motif prediction analysis of genes was performed using the animal TFDB tool (version 4.0).

### 4.7. Construction of lncRNA-mRNA and Protein-Protein Interaction (PPI) Networks

The Search Tool for the Retrieval of Interacting Genes (STRING; version 12.0) was the database used to construct the interactive protein-protein network in the present study. The network was visualized in Cytoscape (version 3.10.1). We generated a lncRNA G8110-centered network in Cytoscape using predicted paired lncRNA-mRNA co-expression data and the interaction results of these mRNAs queried in the STRING database. Our protein-protein interaction (PPI) network was constructed by submitting tested genes to the STRING database and using Cytoscape to visualize PCG interaction networks based on the results.

### 4.8. Statistical Analysis

All experimental data were obtained from at least three independent experiments. Values are shown as the mean ± standard error of the mean (SEM). SPSS (version 19.0) statistics software was used for statistical analysis. Student’s *t*-test was used for individual comparisons. A significance level of < 0.05 was considered to be statistically significant; * *p* < 0.05; ** *p* < 0.01; *** *p* < 0.001.

## 5. Conclusions

In this paper, we have described the identification of a new lncRNA, G8110, from the mesentery adipose tissue of Bama pigs. LncRNA G8110 positively regulated the expression of the *NFE2L1* gene, thereby affecting fat deposition, lipid metabolism, and immune responses in the MAT. These findings indicate that lncRNA G8110 acts as an adaptive mechanism to promote the healthy remodeling of adipose tissue and maintain metabolic homeostasis during obesity. We believe that our discovery of lncRNA G8110 will facilitate further investigations of the molecular mechanisms of fat deposition in pigs.

## Data Availability

In this study, the public RNA-seq and ChIP-seq data of the Bama pig mesenteric adipose tissues for analysis were downloaded from GEO under the BioProject accession code “GSE206539” [21].

## Supplementary Materials

The following supporting information can be downloaded at: https://www.mdpi.com/article/10.3390/ijms242316799/s1.
