# Supplementary material for: Identification of a Novel Long Non-Coding RNA G8110 That Modulates Porcine Adipogenic Differentiation and Inflammatory Responses
Source: Int J Mol Sci. 2023 Nov 27;24(23):16799. doi: 10.3390/ijms242316799 (PMC10706401; doi:10.3390/ijms242316799)
Supplement: Supplementary file 1 [file ijms-24-16799-s001.zip › Supplementary figures .pdf]

Supplementary Figures

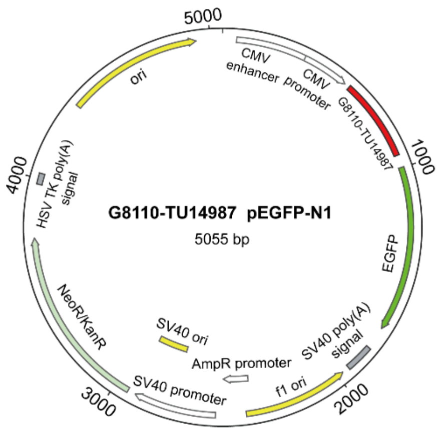

Figure S1. Schematic diagram of the lncRNA G8110 overexpression construct.

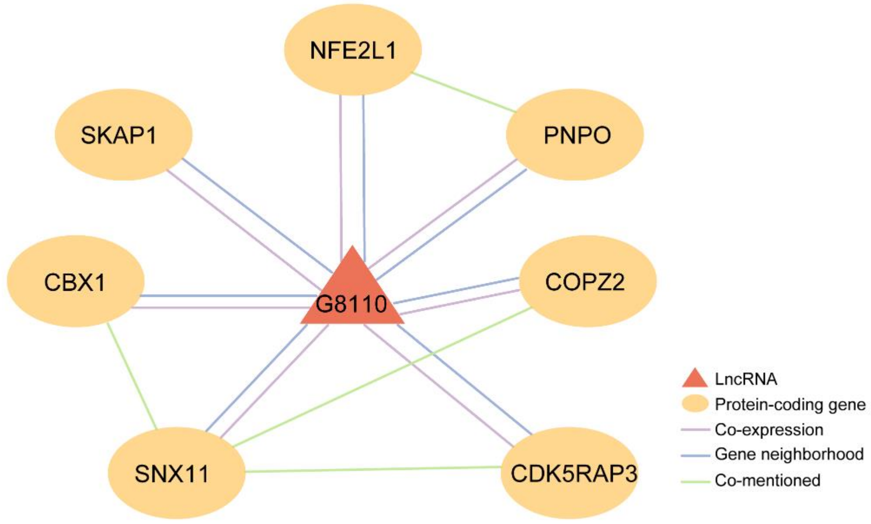

Figure S2. LncRNA–mRNA network. Triangles represent core LncRNA G8110, ellipses represent PCGs, and the lines of various colors represent relationships between genes.

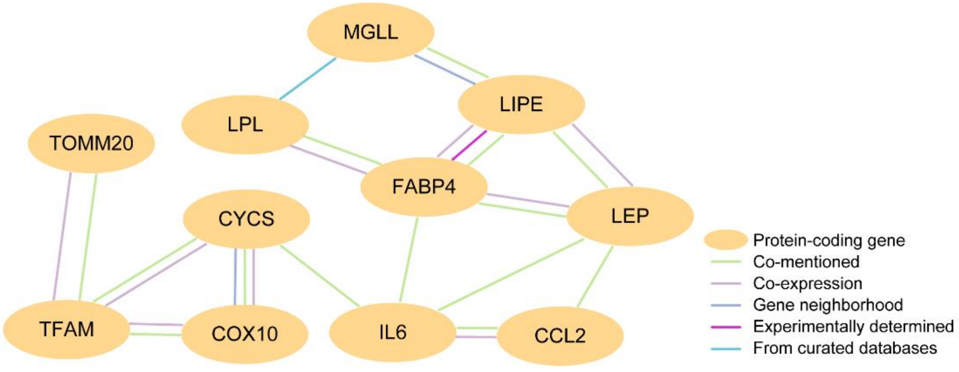

Figure S3. Protein–protein interaction network. Ellipses represent PCGs; the lines of various colors represent relationships between PCGs.

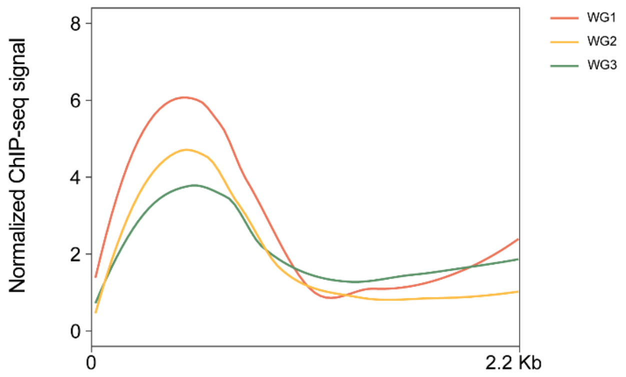

Figure S4. Demonstration of normalized ChIP-seq signals in a 2.2 kb window containing lncRNA G8110.

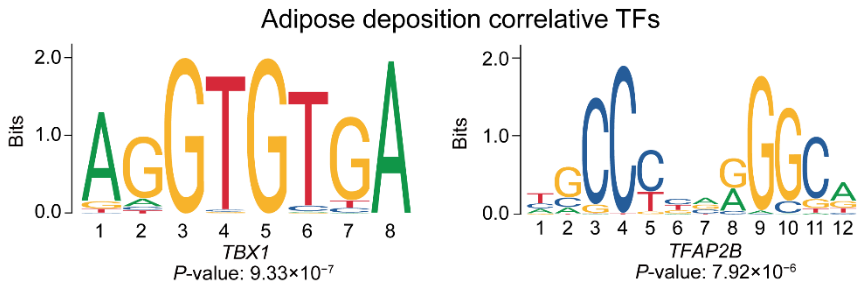

Figure S5. Prediction of a 10 kb region upstream of *NFE2L1* containing TF-binding motifs correlated with adipose deposition.
